# Introgression of Shoot Fly (*Atherigona soccata* L. Moench) Resistance QTLs into Elite Post-rainy Season Sorghum Varieties Using Marker Assisted Backcrossing (MABC)

**DOI:** 10.3389/fpls.2017.01494

**Published:** 2017-09-01

**Authors:** Sunita Gorthy, Lakshmi Narasu, Anil Gaddameedi, Hari C. Sharma, Anuradha Kotla, Santosh P. Deshpande, Ashok K. Are

**Affiliations:** ^1^International Crops Research Institute for Semi-Arid Tropics Hyderabad, India; ^2^Department of Biotechnology, Jawaharlal Nehru Technological University Hyderabad, India

**Keywords:** *Atherigona soccata*, QTLs, marker-assisted backcrossing, introgression, phenotyping, sorghum

## Abstract

Shoot fly (*Atherigona soccata* L. Moench) is a serious pest in sorghum production. Management of shoot fly using insecticides is expensive and environmentally un-safe. Developing host–plant resistance is the best method to manage shoot fly infestation. Number of component traits contribute for imparting shoot fly resistance in sorghum and molecular markers have been reported which were closely linked to QTLs controlling these component traits. In this study, three QTLs associated with shoot fly resistance were introgressed into elite cultivars Parbhani Moti (= SPV1411) and ICSB29004 using marker assisted backcrossing (MABC). Crosses were made between recurrent parents and the QTL donors *viz*., J2658, J2614, and J2714. The F_1_s after confirmation for QTL presence were backcrossed to recurrent parents and the resultant lines after two backcrosses were selfed thrice for advancement. The foreground selection was carried out in F_1_ and BC_n_F_1_ generations with 22 polymorphic markers. Forty-three evenly distributed simple sequence repeat markers in the sorghum genome were used in background selection to identify plants with higher recurrent parent genome recovery. By using two backcrosses and four rounds of selfing, six BC_2_F_4_ progenies were selected for ICSB29004 × J2658, five BC_2_F_4_ progenies were selected for ICSB29004 × J2714 and six BC_2_F_4_ progenies were selected for Parbhani Moti × J2614 crosses. Phenotyping of these lines led to the identification of two resistant lines for each QTL region present on chromosome SBI-01, SBI-07 and SBI-10 in ICSB 29004 and Parbhani Moti. All the introgression lines (ILs) showed better shoot fly resistance than the recurrent parents and their agronomic performance was the same or better than the recurrent parents. Further, the ILs had medium plant height, desirable maturity with high yield potential which makes them better candidates for commercialization. In the present study, MABC has successfully improved the shoot fly resistance in sorghum without a yield penalty. This is the first report on the use of MABC for improving shoot fly resistance in post-rainy season sorghum.

## Introduction

Sorghum is one of the main staple foods for the poor and food insecure people across semi-arid tropics of the world. It is nutritionally superior to other cereals such as rice and wheat with high fiber content, minerals and slow digestibility (Dayakar Rao et al., 2010). It is an important dual-purpose crop grown extensively by resource poor farmers in the states of Maharashtra, Karnataka, Telangana, and Andhra Pradesh in India. It is the lifeline for resource-poor farmers in drylands as it tolerates water deficit stress which is common in the post-rainy season. About 16% of the world’s sorghum is produced in India and the crop is grown in rainy (2–2.5 m ha) and post-rainy seasons (4 m ha) (All India Co-ordinated Sorghum Improvement Project [AICSIP], 2002). The post-rainy season sorghum is prized for its grain and stover quality. Despite good progress in increasing rainy season sorghum productivity over the years (1.2 t ha^-1^), the post-rainy sorghum productivity is quite low (0.8 t ha^-1^). Several biotic and abiotic stresses including terminal moisture stress adversely affect the post-rainy sorghum production. Among the biotic stresses, shoot fly (*Atherigona soccata* Rondani) is the most damaging pest in Asia and also in parts of Africa and Americas restricting the sorghum production (Sharma et al., 2003). Shoot fly is also the major biotic stress on post-rainy season sorghum. About 50% of grain loss has been reported in India by Jotwani (), but sometimes more severe damage up to 90% can occur depending on the shoot fly population (Rao and Gowda, 1967). Over year various methods have been developed for managing the shoot fly and most notable among them is chemical control. Though chemical control is effective, use of chemicals by small farmers is not a feasible option because of their prohibitive cost, limited availability and the toxicity they pose to the environment. Therefore, it is important to develop host–plant resistance (HPR) in sorghum to impart resistance against shoot fly. The cheap and sustainable option for managing shoot fly is the use of resistant cultivars (Sharma et al., 2003; Mohammed et al., 2016).

Sorghum is highly vulnerable to shoot fly damage in the initial stages of crop growth, particularly the late planted crop, in the rainy season. The seedlings are generally attacked by shoot fly in 5–25 days after germination. Generally, the female shoot fly lays single white colored cigar shaped eggs on the lower surface of the newly emerged leaves parallel to the midrib. The larvae, after hatching, crawl to base of the leaf whorl and cut the growing tip, resulting in dead-heart formation (Deeming, 1971). Infestation causes dead-hearts in seedlings as well as in tillers of older plants, resulting in considerable damage to the crop (Sharma et al., 1993). Late own crop is more vulnerable to shoot fly during rainy season, the early-sown crop is more effected during the post-rainy season (Jotwani et al., 1970; Mohammed et al., 2016).

Three components govern shoot fly resistance in sorghum namely non-preference for oviposition, antibiosis, and tolerance (Soto, 1974; Sharma et al., 1997). As mentioned in earlier studies, the main factor for shoot fly resistance is non-preference for oviposition also known as antixenosis (Dhillon et al., 2005b, 2006). Other characters for shoot fly resistance include glossiness, trichomes on both adaxial and abaxial surface of leaves, seedling vigor and epicuticular wax (Sukhani, 1987; Nwanze et al., 1992; Dhillon et al., 2005b, 2006; Kiranmayee et al., 2015). Biochemical parameters such as total chlorophyll content, peroxidase and polyphenol activity also play a significant role in imparting resistance to shoot fly (Singh et al., 2004; Dhillon et al., 2006; Kiranmayee et al., 2015). To identify sources of resistance to shoot fly, a large number of sorghum germplasm accessions were screened and resistance sources were identified (Sharma et al., 1992; Kumar et al., 2014). Using these resistance sources in crossing program and selection for resistance in screening blocks, number of shoot fly resistant sorghum varieties, parents and hybrids were developed and commercialized (Kumar et al., 2008). However, use of conventional breeding methods to develop elite cultivars with resistance to insect pest is often time consuming and laborious. Also, resistance to insects is a quantitatively inherited trait which relies on the environmental condition, hence, it becomes difficult to achieve an appreciable increase in resistance against any insect pest (Tao et al., 2003; Riyazaddin et al., 2016). To overcome such problems, molecular breeding techniques have been deployed in many crop species (Kulwal et al., 2011). Further studies on shoot fly resistance mechanisms identified reduced dead hearts incidence, reduced oviposition incidence, improved leaf glossiness, higher trichomes on the abaxial surface of the leaf as component traits to select for shoot fly resistance in sorghum (Dhillon et al., 2005b; Anandan et al., 2009). The biotechnological approaches to address the traits/mechanisms for improving shoot fly resistance were more promising (Kiranmayee et al., 2015). Quantitative trait loci (QTL) for the component traits imparting shoot fly resistance had been identified on chromosome SBI-01 [Oviposition Non-preference 28 days after seedling emergence (DAE) QEg28.dsr-1.1, Seedling vigor QSv, Trichome density on lower leaf surface QTdl.dsr-1.1, Glossiness QSv.dsr-1.1, QGs.dsr-1], SBI-05 (Glossiness, Oviposition Non-preference and less dead hearts), SBI-07 (Glossiness QGs.dsr-7, Oviposition Non-preference on 21 and 28 DAE QEg21.dsr-7; QEg28.dsr-7 and dead-hearts Qdh.dsr-7.1; Qdh.dsr-7.2) and SBI-10 (Insect resistance, Glossiness QGs.dsr-10, Oviposition Non-preference on 21 and 28 DAE QEg21.dsr-10; QEg28.dsr-10 and dead hearts Qdh.dsr-10.1; Qdh.dsr-10.2; Qdh.dsr-10.3; Qdh.dsr-10.4, Leaf Trichomes on upper and lower leaf surface Tdl, Tdu, QTdu.dsr-10.1; QTdl.dsr-10.1; QTdu.dsr-10.2; QTdl.dsr-10.2 and Seedling vigor QSv.dsr-10) (Folkertsma et al., 2003; Deshpande et al., 2006; Satish et al., 2009). All these studies utilized a common shoot fly resistance donor germplasm line *viz*., IS18551, originated from Ethiopia. These QTLs were introgressed into elite sorghum maintainer lines, *viz*., 296B and BTx623, using marker-assisted backcrossing (Deshpande et al., 2010). The effects of these introgressed QTLs on the shoot fly resistance were confirmed by field-level evaluation of several versions of introgression lines (ILs) for each QTL per genetic background over multiple seasons. For each QTL, most stable versions, i.e., J2658-6, J2698-7 from SBI- 01, J2714-3, J2743-3 from SBI-07, J2614-3, J2614-5 from SBI-10 and J2833-11, J2799 from SBI-05 of introgressions, confirmed for presence of shoot fly resistance alleles from donor line IS18551 in BTx623-background (a shoot fly susceptible, elite B-line) were used as donors. However, none of these ILs are in adapted to major sorghum growing areas. Therefore, in the study, we have undertaken introgression of QTLs controlling shoot fly resistance component traits (oviposition non-preference, seedling vigor, glossiness, dead hearts percent), present on three different chromosomes *viz*., SBI-01, SBI-07 and SBI-10 into the elite post-rainy season sorghum varieties using marker assisted backcrossing (MABC).

## Materials and Methods

### Parent Materials

Two elite sorghum genotypes *viz*., SPV1411 (Parbhani Moti) and ICSB 29004 were used as recurrent parents for introgression of shoot fly resistance QTLs. Parbhani Moti, a popular variety with farmers released by Vasantrao Naik Marathwada Krishi Vidyapeeth, Parbhani, Maharashtra, India. It has bold and pearl like grains with excellent grain and fodder quality and can tolerate drought. ICSB 29004 is a high yielding B-line developed at ICRISAT-Patancheru and used by public and private sector organizations in hybrids development. After screening several sets of MABC derived ILs under field conditions for shoot fly resistance (Deshpande et al., 2010), three BC_4_F_2_-derived ILs *viz*., J2658-6, J2714 and J2614, carrying shoot fly resistance QTLs on chromosomes SBI-01, SBI-07, and SBI-10, respectively, were used as donor parents in the crossing program. These donors are essentially ILs of BTx623, an elite sorghum line from the United States but susceptible to shoot fly into which the shoot fly resistance QTLs have been introgressed. The four validated QTLs imparting shoot fly resistance on chromosome SBI-01, SBI-07 and SBI-10 used in this study govern different component traits, such as chromosome SBI-01 Oviposition Non-preference and Seedling vigor; SBI-07 Glossiness and Oviposition Non-preference, and SBI-10 Glossiness, Oviposition Non-preference, Dead hearts, and Leaf Trichomes.

### Molecular Markers

In the present study we used molecular markers to confirm the presence of the target QTL in backcross progenies. In the absence of information on exact location of the target, each chromosomal segment was checked by at least three markers in foreground selection (FGS): one near the QTL and the two others at right- and left-hand sides or flanking QTL region. Markers were selected to delimit a zone of approximately 10 Mb around QTL for cross ICSB 29004 × J 2658, approximately 6 Mb for cross ICSB29004 × J2714 and SPV 1411 × J2614. Background selection for non-carrier chromosomes was carried out with two or more markers on each chromosome.

### Genotyping

#### Deoxyribonucleic Acid (DNA) Extraction

DNA extraction was carried out using modified Cetyl Tri-methyl Ammonium Bromide (CTAB) extraction method. DNA extraction was done from leaves of 20 days old seedlings of parents, F_1_’s, and backcrossed lines using the modified CTAB method (Mace et al., 2003). DNA was further treated with RNase to remove any RNA contamination followed by purification of DNA with phenol/chloroform/iso-amyl alcohol (25:24: 1). Lastly, DNA was precipitated using chilled ethanol (Mace et al., 2003). DNA was quantified on 0.8% agarose gel and concentration was normalized to ∼5 ng μL^-1^.

#### Marker Genotyping Using Polymerase Chain Reaction (PCR)

Simple sequence repeat (SSR) markers from target genomic region reported in earlier studies were used for assessing parental polymorphism (**Table 1**). The polymorphic markers identified in parental polymorphism screening were used for FGS for each QTL. For background screening markers from the whole sorghum genome were selected. In all these steps, the SSRs were taken and polymerase chain reactions were performed in 5 μl volumes using PCR system PE9700 thermal cycler (Applied Biosystems, United States) with touchdown program. Reaction conditions were as follows: initial denaturation for 15 min at 94°C, followed by 10 cycles of denaturation for 10 s at 94°C, annealing at 61–52°C for 20 s, the annealing temperature for each cycle is reduced with 1°C and extension at 72°C for 30 s and 35 cycles of denaturation for 10 s at 94°C, annealing at 54°C for 20 s and extension at 72°C for 30 s. Lastly, 20 min extension time at 72°C so that both the DNA strands amplify to equal length. PCR products of 2–4 primer pairs amplifying SSRs were pooled on basis of product size and dye/color for each QTL across donor-recurrent parent combination. These PCR products were pooled with 0.5 μl of VIC, and 1.0 μl each of FAM, NED, and PET dyes with 7.0 μl Hi-Di Formamide and 1.5 μl milliQ water to a final volume 12.0 μl per reaction. These pooled products were denatured at 94°C for 5 min. The denatured products were then subjected to capillary electrophoresis using ABI Prism 3700*xl* DNA Sequencer. Analysis was done using GeneMapper software of Applied Biosystems, (Carlsbad, CA, United States).

**Table 1:** List of SSR Markers used for polymorphism study for different shoot fly resistant QTLs.

QTLs	SSRs tested for polymorphism		Polymorphic SSRs	
QTLs on	6	Xtxp329	5	Xtxp329
SBI-01 (LG A)		Xtxp149		Xtxp149
		Xtxp088		Xisep1035
		Xisep1035		Xisep1028
		Xisep1028		Xtxp075
		Xtxp075		
QTLs on	18	Xtxp417	6	mSFC107
SBI-07 (LG E)		Xtxp413		mSFC106
		mSFCILP85		mSFCILP94
		Xtxp481		mSFC112
		Xtxp093		Xtxp159
		mSFC107		Xtxp278
		mSFCILP88		
		mSFC105		
		mSFC106		
		mSFCILP93		
		mSFC110		
		mSFCILP94		
		mSFC111		
		mSFC112		
		mSFC113		
		mSFC114		
		Xtxp159		
		Xtxp278		
QTLs on	12	Xisep0634,	11	Xisep0634
SBI-10 (LG G)		Xgap001		Xgap001
		Xnhsbm1008		Xnhsbm1008
		Xsbarslbk10.06		
		Xnhsbm1011		Xnhsbm1011
		Xisep0643		Xisep0643
		Xtxp320		Xtxp320
		Xisep0639		Xisep0639
		msbCIR227		msbCIR227
		Xcup16		Xcup16
		Xtxp141		Xtxp141
		Xcup07		Xcup07

#### Developing Backcrossed Lines and Foreground Screening

**FIGURE 1:**
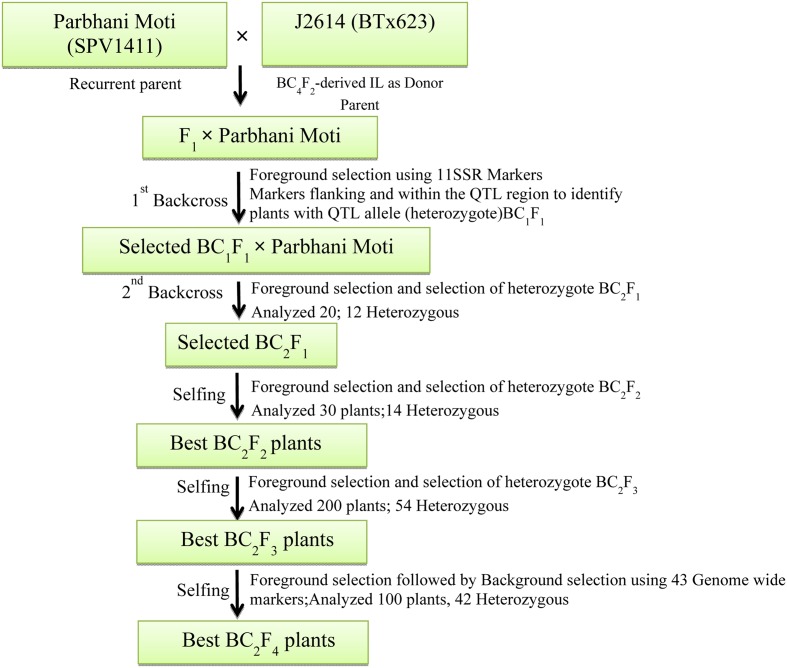
Breeding scheme used for the development of shoot fly resistant backcross lines for the cross P. Moti × J2614. Similar pattern was followed for other crosses ICSB29004 × J2658 and ICSB29004 × J2714.

The three crosses (Parbhani Moti × J2614, ICSB29004 × J2658, and ICSB29004 × J2714) made in the study were executed in a plant × plant crossing mode for the developing the ILs. The F_1_’s generated between the donor and recipient (recurrent) parents were further backcrossed with the recurrent parents (SPV1411 = Parbhani Moti and ICSB 29004) to produce BC_1_F_1_s. To have synchronization, the parents (eight seeds) were staggered with 1 week difference in two sowing dates wherein F_1_’s (six seeds) were sown in the second sowing date. This staggered sowing ensured nicking of F_1_s and corresponding recurrent parents to undertake backcrossing. Emasculation of recurrent parents was done for the generation of F_1_ seeds and backcrosses were made using standard schemes (**Figure 1**). After confirming the hybridity of the F_1_ plants using foreground markers (**Table 2**), true heterozygous plants were backcrossed with their respective recurrent parents to generate BC_1_F_1_ seeds. The markers used for undertaking background selection were selected from published information (**Table 3**). The number of heterozygous plants identified in each generation was documented (**Table 4**). After undertaking two rounds of back-crossing, the selected plants were selfed for three generations (BC_2_F_4_) to make the plants homozygous as well as to multiply the seed of ILs. For FGS, the complete genome genotyping was performed by using SSR markers distributed over all 10 sorghum chromosomes. In each generation, plants showing the heterozygous alleles for different shoot fly resistance component traits were selected. At the final stage, 17 homozygous lines carrying target loci along with similarity with recurrent parents’ genome were selected in BC_2_F_4_ generation for shoot fly resistance screening.

**Table 2:** Details of SSR markers used for undertaking foreground selection in marker-assisted backcrossing program for transferring QTLs for shoot fly resistance.

QTL containing Chromosome number/Linkage Group	Marker names	Forward primer sequence (5′–3′)	Reverse primer sequence (5′–3′)	Motif	Reference
**SBI-01/ LG A**	Xtxp329	CACGACGTTGTAAAACGACACTAC GAAGGTGTTTAGTTTAAGGG	CATTCATAAAACTAAA CGAAAAACG	(ATC)8 + (CTT)22	Bhattramakki et al., 2000
	Xtxp149	AGCCTTGCATGATGTTCC	GCTATGCTTGGTGTGGG	(CT)10	Bhattramakki et al., 2000
	Xisep1035	CACGACGTTGTAAAACGACC ACTTTCTACCGCTCCTTCG	AGTGATGATGATGACCGAACC	TGAT(5)	ICRISAT_Ramu, unpublished
	Xisep1028	CACGACGTTGTAAAACGACCAG CGACCATGAGGATGAC	TGGCATGCATCAAACAAGAT	GCA(4)	ICRISAT_Ramu, unpublished
	Xtxp075	CGATGCCTCGAAAAAAAAACG	CCGATCAGAGCGTGGCAGG	(TG)10	Bhattramakki et al., 2000
**SBI-07/ LG E**	mSFC107	CCTCCTGATCCATTTTGCTG	CATGCTTCATGCTTTGACCA	(TA)6	Satish et al., 2012
	mSFC106	GAGGTGTCGTGGATTTGACC	CCCGTAAGCAGGCCATAGTA	(GA)7	Satish et al., 2012
	mSFCILP94	GAGCCTCAGTTCGATTCTGG	CCGGAAGAGGCGATAAAGA	IN2	Satish et al., 2012
	mSFC112	TATTGCTGCTGTCCTGTTGG	CATCCAAAGGGGCCTTTATT	(AT)7	Satish et al., 2012
	Xtxp159	ACCCAAAGCCCAAATCAG	GGGGGAGAAACGGTGAG	(CT)21	Bhattramakki et al., 2000
	Xtxp278	CACGACGTTGTAAAACGACGGGT TTCAACTCTAGCCTACCGAACTTCCT	ATGCCTCATCATGGTT CGTTTTGCTT	(TTG)12	Bhattramakki et al., 2000
**SBI-10/ LG G**	Xisep0634	CACGACGTTGTAAAACGACGCA TAGCCACCAGATCTTCC	AATCATGCTTGCACACTTGC	CAG(5)	ICRISAT_Ramu, unpublished
	Xgap001	TCCTGTTTGACAAGCGCTTATA	AAACATCATACGAGCTC ATCAATG	(AG)16	Brown et al., 1996
	Xnhsbm1008	TGAATGGCAATGTGTTTGGT	CGTGTTCCCGTAGGTTGTC	(TCTA)18	Satish et al., 2009
	Xnhsbm1011	TGGGATGCCATATTCTTTTTG	GTTCCTGGTGTTCGTTTGCT,	(TTC)17	Satish et al., 2009
	Xisep0643	CACGACGTTGTAAAACGACC TCACCTTGGGAGCTGAATC	GGAGGACCTAGCAAGCAAGA	TC(7)	ICRISAT_Ramu, unpublished
	Xtxp320	TAAACTAGACCATATACTGC CATGATAA	GTGCAAATAAGGGCT AGAGTGTT	(AAG)20	Bhattramakki et al., 2000
	Xisep0639	CACGACGTTGTAAAACGACTCGGA CGGAGTCATCAGATA	GCCTTCGTGTCTTCTGTCCT	TCT(6)	ICRISAT_Ramu, unpublished
	msbCIR227	CACGACGTTGTAAAACGACTTC ACTGTCAAAACTTGGAA	TGAATAATATCTTGCA TATACGTG		Mace et al., 2009
	Xcup16	TGCAGTGCTAGCTCATGGTC	CTTTCCAGCCTCCCATATCC	(CTTTT)4	Schloss et al., 2002
	Xtxp141	TGTATGGCCTAGCTTATCT	CAACAAGCCAACCTAAA	(GA)23	Bhattramakki et al., 2000
	Xcup07	CTAGAGGATTGCTGGAAGCG	CTGCTCTGCTTGTCGTTGAG	(CAA)8	Schloss et al., 2002

**Table 3:** Details of SSR markers used for undertaking background selection.

S No	Locus name	Product	Chromosome	Physical Map Position (Mb)	Reference
1	Xisep0728	188	SBI- 01	12.2	Ramu, 2009
2	Xgpsb089	168	SBI-01	43.9	Agropolis-Cirad-Genoplante, unpublished
3	Xtxp320	289	SBI-01	55.4	Bhattramakki et al., 2000; Kong et al., 2000
4	XmSbCIR306	121	SBI-01	71.0	Agropolis-Cirad-Genoplante, unpublished
5	Xtxp075	172	SBI-01	60.2	Bhattramakki et al., 2000; Kong et al., 2000
6	Xisep1145	175	SBI 02	2.0	Ramu, 2009
7	XmSbCIR223	115	SBI 02	4.7	Agropolis-Cirad-Genoplante, unpublished
8	Xtxp072	122	SBI 02	27.9	Bhattramakki et al., 2000; Kong et al., 2000
9	Xtxp001	211	SBI 02	61.4	Bhattramakki et al., 2000; Kong et al., 2000
10	Xgap084	170-190	SBI 02	63.2	Brown et al., 1996
11	Xcup11	164	SBI 03	2.0	Schloss et al., 2002
12	Xisep0107	198	SBI 03	3.2	Ramu, 2009
13	Xisep0101	210	SBI 03	7.1	Ramu, 2009
14	Xisep132	201	SBI 03	16.2	Ramu, 2009
15	Xisep0114	193	SBI 03	51.2	Ramu, 2009
16	Xtxp031	221	SBI 03	55.2	Bhattramakki et al., 2000; Kong et al., 2000
17	Xisep0202	184	SBI 04	4.8	Ramu, 2009
18	Xisep0203	210	SBI 04	10.0	Ramu, 2009
19	Xtxp012	192	SBI 04	48.6	Bhattramakki et al., 2000; Kong et al., 2000
20	Xgap010	250	SBI 04	51.7	Brown et al., 1996
21	Xtxp327	156	SBI 04	59.3	Bhattramakki et al., 2000; Kong et al., 2000
22	Xtxp021	178	SBI 04	68.0	Bhattramakki et al., 2000; Kong et al., 2000
23	mSbCIR248	90	SBI-05	4.7	Agropolis-Cirad-Genoplante, unpublished
24	Xisep1133	193	SBI-05	17.5	Ramu, 2009
25	Xtxp136	240-243	SBI-05	57.5	Bhattramakki et al., 2000; Kong et al., 2000
26	Xtxp006	119	SBI 06	3.2	Bhattramakki et al., 2000; Kong et al., 2000
27	Xtxp145	238	SBI 06	49.3	Bhattramakki et al., 2000; Kong et al., 2000
28	Xtxp278	248	SBI-07	51.1	Bhattramakki et al., 2000; Kong et al., 2000
29	SbAG-B02	117	SBI-07	62.5	Taramino et al., 1997; Agrama et al., 2002
30	mSbCIR300	109	SBI-07	58.3	Agropolis-Cirad-Genoplante, unpublished
31	Xtxp417	177	SBI-07	1.5922	Bhattramakki et al., 2000; Kong et al., 2000
32	Xtxp321	205	SBI 08	50.5	Bhattramakki et al., 2000; Kong et al., 2000
33	gpsb123	288-296	SBI 08	52.2	Agropolis-Cirad-Genoplante, unpublished
34	Xtxp273	223	SBI 08	0.2	Bhattramakki et al., 2000; Kong et al., 2000
35	Xtxp287	364	SBI 09	4.2	Bhattramakki et al., 2000; Kong et al., 2000
36	Xisep1241	182	SBI 09	8.4	Ramu, 2009
37	Xtxp10	144	SBI 09	47.9	Bhattramakki et al., 2000; Kong et al., 2000
38	sb5-206	125	SBI 09	59.2	Brown et al., 1996
39	mSbCIR283	142	SBI-10	18.1	Agropolis-Cirad-Genoplante, unpublished
40	Xtxp290	265	SBI-10	49.29	Bhattramakki et al., 2000; Kong et al., 2000
41	Xnhsbm1011	151	SBI-10	54.9084	Satish et al., 2009
42	mSbCIR262	213	SBI-10	55.3	Agropolis-Cirad-Genoplante, unpublished
43	Xtxp141	162	SBI-10	58.2	Bhattramakki et al., 2000; Kong et al., 2000

**Table 4:** Details of number of plants analyzed and selected in each back cross generation confirmed through foreground and background screening and percentage recovery of recurrent parent genome.

Crosses	Number of plants analyzed and heterozygotes in each generation										Number of plants after BGS (with % RPG recovery)
	BC_1_F_1_		BC_2_F_1_		BC_2_F_2_		BC_2_F_3_		BC2F4		
	Analyzed	Heterozygous	Analyzed	Heterozygous	Analyzed	Heterozygous	Analyzed	Heterozygous	Analyzed	Heterozygous	
ICSB29004 × J2658	25	9	30	11	200	78	100	44	100	35	6 (80–92%)
ICSB29004 × J2714	21	5	30	11	200	96	100	32	100	32	5 (80–90%)
Parbhani Moti × J2614	20	12	30	14	200	54	100	42	100	41	6 (80–90%)

#### Markers Used for Background Screening

Except for target locus, the entire genomic region was selected for background screening using recurrent parent marker alleles. In our study, 100 SSR markers were screened for background selection with known chromosomal positions spanning all 10 chromosomes. Forty-three out of 100 SSRs were identified as polymorphic and were further used for screening the ILs having maximum recurrent parent genome (**Table 3**). Polymerase chain reaction was set up with all the 43 SSR markers and the bands were scored on 4% agarose gel. The scores were then run in Graphical Genotypes (GGT) version 2.0 for the assessment of the recovery of the recurrent parent genome.

### Field Evaluation of Plant Materials

The 17 BC_2_F_4_ ILs from three crosses developed in this study were evaluated along with their parents and checks (shoot fly resistant check IS18551 and susceptible checks Swarna and 296B) in replicated trials in two test environments *viz.*, 2014 post-rainy and 2015 rainy season at ICRISAT, Patancheru, Hyderabad, India (altitude 545 m above mean sea level, latitude 17.530 N and longitude 78.270 E) in both breeding block and shoot fly screening block. Randomized complete block design (RCBD) was used to conduct field evaluation. The seeds were planted in three replications in a two-row plot of 2 m with 75 cm of spacing between the rows. Thinning of the 10 days old seedlings was done and the spacing between the plants were maintained 15 cm. High shoot fly population was maintained using the inter-lard fish meal technique (Soto, 1974) to conduct field evaluations. Except plant protection measures all the agronomic practices were followed to raise the crop in the shoot fly screening block.

### Phenotyping for Shoot Fly Resistance

The data on five component traits contributing to shoot fly resistance were recorded for two seasons *viz.*, 2014 post-rainy and 2015 rainy, at ICRISAT-Patancheru, Hyderabad, India. The component traits recorded in the field trial were leaf surface glossiness (GS), trichome density on adaxial (upper; TDU) and abaxial (lower; TDL) leaf surfaces, seedling vigor (SV), oviposition at 28 DAE (EG28) and dead hearts percentage (DH%). Leaf glossiness was visually scored in the morning hours at 12 DAE on a scale of 1–5 scores with 1 being glossy (light green, shiny, narrow, and erect leaves), and 5 -non-glossy (dark green, dull, broad, and drooping leaves). Seedling vigor was recorded visually at 14 DAE on a 1–5 scale, where 1 stands for low vigor (plants showing minimum growth, less leaf expansion) and 5 for high vigor (plants showing maximum height, full leaf expansion, and robustness). Oviposition non-preference is the number of eggs laid on each seedling from each plot at 14, 21, and 28 DAE. The overall shoot fly damage was calculated by taking the percentage of dead hearts (DH%) on 14, 21, and 28 DAE by using the formula (ratio of the number of dead-hearts/total number of plants × 100). To record the trichome density both trichomes on the adaxial leaf surface (TDU) and abaxial surface (TDL) were recorded at 14 DAE. For this, the central portion of the fifth leaf counting from base from three random seedlings were taken and cut into 2 cm^2^ approximately size and were placed in acetic acid: alcohol (2:1) solution. Later the leaf segments were transferred to 90% lactic acid in small vials (Maiti et al., 1980). The trichomes on both abaxial and adaxial leaf surface were counted by placing the leaf segments on a slide and observed under the 10× microscopic field and expressed as a number of trichomes/microscopic field (no./mm^2^).

### Statistical Analysis

All the experiments were carried out with three biological replicates. Analysis of variance was calculated using GenStatR13th version and *F*-test was performed to identify the significant difference among genotypes. Least significant difference (LSD) at *P* ≤ 0.05 was used to calculate the treatment means. The standard error bars were used to represent the standard error among different ILs.

### Agronomic Performance of the Selected BC_2_F_4_ Lines

Data for agronomic performance for above-mentioned crosses and their parents was recorded in the breeding block for two seasons (2014 post-rainy and 2015 rainy) in a RCBD in three replications in black soil at the ICRISAT-Patancheru, India (latitude17.53°N, longitude78.27°E, and altitude of 545 m). Agronomic parameters such as time to 50% flowering (days), plant aspect score for agronomic desirability (on a scale of 1 to 5, where 1 – most desirable and 5 – least desirable), 100-grain weight and grain yield per plot were scored.

## Results

### Development of MABC Introgression Lines

To introgress the QTLs located on chromosomes SBI-01, SBI-07, and SBI-10 conferring resistance to shoot fly, three different donors *viz*., J2658, J2714, and J2614 (shoot fly resistant), were crossed separately with recurrent parents *viz*., ICSB 29004 and Parbhani Moti (SPV1411) to generate F_1_ seeds. After confirmation of hybridity using SSR markers, the heterozygous plants were used for further backcrossing. Sorghum SSR markers were used for foreground and background selection in the backcrossed progenies. In this study, homozygous lines were developed by undertaking two backcrosses and three selfings.

Two elite sorghum lines ICSB 29004 and Parbhani Moti (recurrent parents) were used as female parents and crossed with J2658, J2714, and J2614 (donor parents carrying shoot fly resistance QTLs) as male parents. This resulted in the generation of 25, 21 and 20 F_1_ seeds from crosses ICSB 29004 × J2658, ICSB29004 × J2714, and Parbhani Moti × J2614, respectively. Out of which, nine (ICSB 29004 × J2658) five (ICSB29004 × J2714) and 12 (Parbhani Moti × J2614) true hybrids were identified with the help of polymorphic markers. These true F_1_s were used to make the first backcross, BC_1_F_1_ with their respective recurrent parents. In each backcross generation, based on phenotypic similarity with recurrent parent few plants were selected and analyzed for heterozygosity (**Table 4**). Further, the DNA was isolated and FGS was done with 5 (ICSB 29004 × J2658), 6 (ICSB29004 × J2714), and 11 (Parbhani Moti × J2614) SSR markers. Based on the FGS results, four plants that are heterozygous for all markers were selected for second round of backcrossing to generate BC_2_F_1_ seeds.

### Molecular Markers for Foreground Selection

For introgression of three shoot fly resistant QTLs, parental polymorphism was carried out between the donor lines and elite recurrent parents using 58 SSRs distributed across genomic region of our target QTLs on linkage groups SBI-01 (LG-A), SBI-07 (LG-E), and SBI-10 (LG-G) (**Table 1**). Thirty-three out of 58 SSRs were found to be polymorphic and were used for FGS across donor-recurrent parent combination for each QTL. The differences in allele size among parents varied between 3 and 100 bp. These polymorphic markers were used to identify and select the QTL region in segregating generations (**Table 2**).

Genomic regions of three QTLs on SBI-01, SBI-07, and SBI-10 contributes up to a phenotypic variation of 11.5, 18.3, and 20%, respectively (Deshpande, 2005; Satish et al., 2009, 2012). It was reported that Cysteine protease Mir1 protein is the major insect resistance gene in sorghum and the same gene is also responsible for insect resistance in maize (Satish et al., 2009, 2012). Cysteine protease Mir1 gene is identified on SBI-10 and was found to be highly associated with shoot fly resistance component traits such as glossiness, dead hearts percent, trichome density, etc. Therefore, more emphasis was laid on QTL present on LG G, i.e., on SBI-10 in this study. Different number of polymorphic markers were used for the identification of QTLs from three chromosomes SBI-01, SBI-07 and SBI-10 (**Table 1**). But only four markers including flanking and target specific markers were employed in the backcross generations.

### Marker Assisted Foreground Selection

Crosses were made between recurrent and donor parents, to yield three cross combinations *viz*., ICSB 29004 × J 2658, ICSB29004 × J2714 and Parbhani Moti × J2614 during 2011 post-rainy season. Each cross produced 45, 24 and 26 F_1_ seeds, respectively. The heterozygous F_1_ plants were screened with foreground markers to identify plants carrying both donor and recurrent parent alleles using capillary electrophoresis and analyzed using gene mapper software. The plants having alleles from both the genotypes were backcrossed to the recurrent parent during 2012 rainy season to generate the BC_1_F_1_ seed. The BC_1_F_1_ plants were screened for heterozygous allele followed by identification of progenies with maximum similarity with the recurrent parent 2012 post-rainy season. The allele size in base pairs (bp) for the linked markers used for both the parents ICSB 29004 and Parbhani Moti showed clear between parental types and heterozygotes (**Table 5**). The best plants of BC_1_F_1_ having phenotypic similarity with recurrent parents and carrying the target gene were again crossed with the recurrent parent to generate BC_2_F_1_ seed and 10 plants of the BC_2_F_1_ generation were selected in 2013 rainy season. Similarly, the BC_2_F_1_ plants were screened to identify the plants in heterozygous form in 2013 post-rainy season. Three rounds of selfing was performed to generate BC_2_F_4_ generation. All the crosses were undertaken simultaneously in a similar manner. In the present study QTL flanking as well as QTL specific markers were used to identify the heterozygosity in each backcross progeny. Hence, we considered that introgression of QTL was successfully done with a size of approximately 10 Mb region introgressed in the cross ICSB 29004 × J2658, approximately 6 Mb in the cross ICSB29004 × J2714 and approximately 6 Mb in the cross Parbhani Moti × J2614. The differences between heterozygotes and their respective recurrent and donor parents were obvious.

**Table 5:** Allele size in base pairs (bp) for the linked markers used for two parents ICSB 29004 and Parbhani Moti.

QTLs for shoot fly Resistance	Chromosome Number	Markers spanning the QTL region	Physical map position (Mb)	Parbhani Moti	ICSB29004
QTL A	SBI 01	Xtxp329	50.1325	189	153
		Xtxp149	50.7111	161	158
		Xisep1035	51.0680	151	163
		Xisep1028	52.0574	211	217
		Xtxp075	60.2630	172	180
QTL E	SBI 07	XnhsbmSFC107	45.2	191	195
		XnhsbmSFC106	43.7	170	170
		XnhsbSFCILP94	48.1	377	384
		XnhsbmSFC112	57.1	176	176
		Xtxp159		181	185
		Xtxp278	51.1	247	253
QTL G	SBI 10	Xisep0634	54.4	222	223
		Xgap001	54.5	260	260
		Xnhsbm1008	54.7	′195.95/208.03	224
		Xnhsbm1011	54.9	′185.65/193.65	188
		Xisep0643	55.01	218	218
		Xtxp320	55.3	′280.28/288.42	–
		Xisep0639	55.6	211	206
		msbCIR227	55.7	118	122
		Xcup16	57.7	240	249
		Xtxp141	58.2	′142.97/158.89	172
		Xcup07	60.5	211	–

### Marker Assisted Background Selection

**FIGURE 2:**
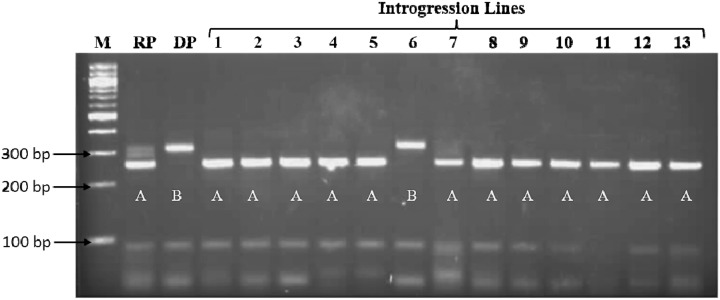
Background screening of introgression lines using the PCR product of marker Xgap10 on 4% agarose gel. M: 100bp ladder, RP: Recurrent Parent band (∼250 bp), DP: Donor Parent band (∼300 bp). The recurrent parent band was scored as A, donor parent band was scored as B.

**FIGURE 3:**
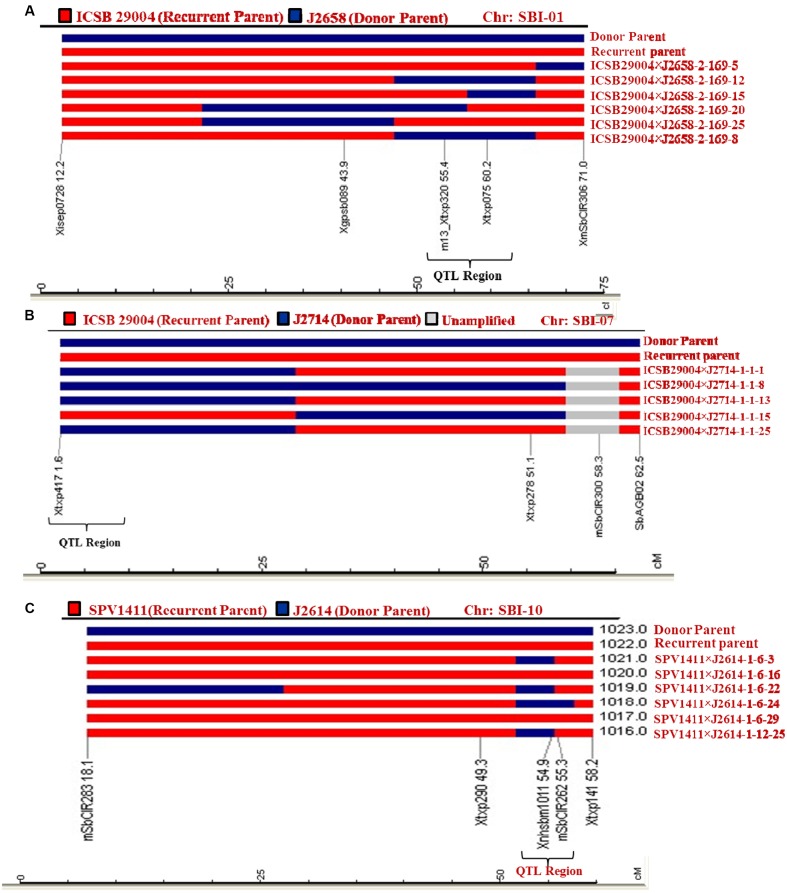
Graphical genotypes of selected lines using SSR markers for the carrier chromosomes for marker-assisted backcrossing lines for resistance to shoot fly **(A)** Polymorphic SSR markers on the carrier chromosome (SBI-01) between parental lines (ICSB 29004 × J2658) were used to analyze the introgression of donor parent genome associated with resistance QTL region. **(B)** Graphical genotypes (GGT) were generated after genotyping MABC lines for QTL on SBI-07 with specific markers that showed polymorphism between ICSB 29004 and J2714. **(C)** MABC lines for QTL SBI- 10 region were genotyped with specific markers that showed polymorphism between Parbhani Moti (SPV1411) and J2614. The genotyping data was used for preparation of GGT. In each case, GGT identified the plants with minimum amount of the donor parent genome.

Heterozygotes from BC_2_F_4_s developed from the crosses ICSB 29004 × J2658, ICSB29004 × J2714 and Parbhani Moti × J2614 were subjected to background screening using 42 SSR markers. The PCR bands were scored on 4% agarose gel (**Figure 2**). In the gel, the recurrent parent was scored as A, donor parent was scored as B, unamplified as U and heterozygous as H. The results showed that the introgressed lines have more than 80% recurrent genome content. A graphical representation of the QTL carrier chromosome SBI-01, chromosome SBI-07 and chromosome SBI-10 of the selected improved lines indicated that maximum recovery of the recurrent parent genome was found out to be 91.6% in the cross ICSB 29004 × J 2658, followed by 88.9% in the cross ICSB29004 × J2714 and 87.5% in Parbhani Moti × J2614 (**Figures 3A–C**). Some of the chromosomes carrying the trait of interest, i.e., SBI-01, SBI-07, and SBI-10 had residual segments from donor genome.

### Phenotyping of Introgression Lines for Shoot Fly Resistance

All selected 17 BC_2_F_4_ progenies obtained from three crosses *viz*. ICSB 29004 × J 2658, ICSB29004 × J2714 and Parbhani Moti (SPV 1411) × J2614 along with the recurrent parents and checks were screened for shoot fly resistance-conferring component traits *viz*., glossiness (7 DAE), seedling vigor (7 DAE), oviposition, trichome density (12 DAE), and dead-hearts percentage (28 DAE).

### Phenotyping of Introgression Lines from the Cross ICSB 29004 × J 2658

**FIGURE 4:**
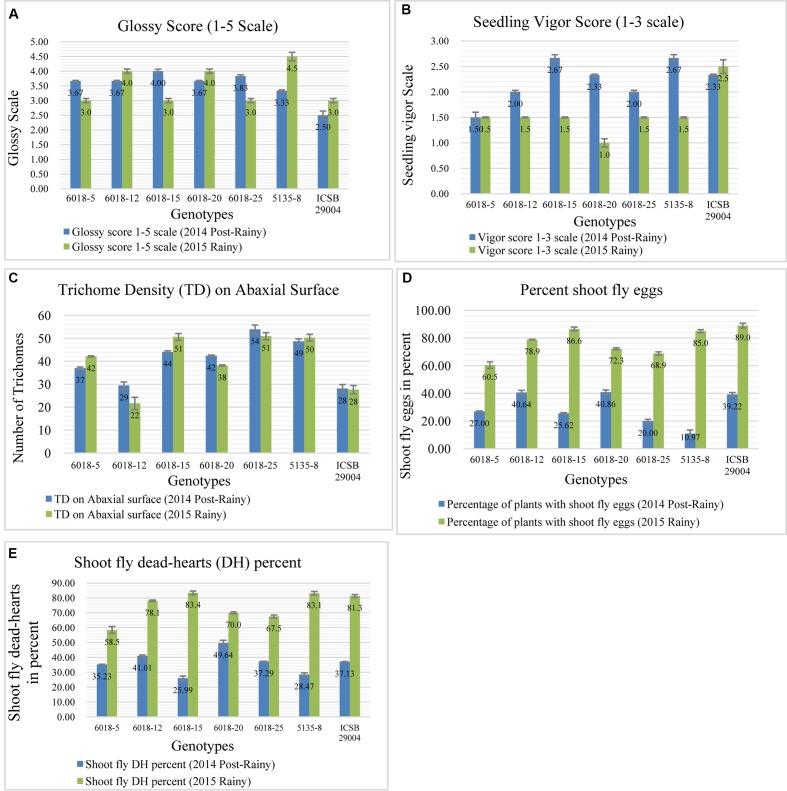
Performance of different introgression lines (1–6) along with their recurrent parent (ICSB 29004) for different shoot fly resistance component traits in two screening seasons viz., 2014 Post-rainy as well as in 2015 Rainy. **(A)** Comparison of Glossy score of among different progenies with recurrent parent, **(B)** Progenies showing higher seedling vigor compared to recurrent parent, **(C)** Comparison of trichome density for the progenies compared to the recurrent parent, **(D)** Oviposition percent of progenies compared to recurrent parent, **(E)** Overall dead-heart percentage for two screening seasons. Bars represents standard error. Significance was determined at *P* < 0.05.

From chromosome SBI-01 we introgressed a region between 50.13 (mega bases) and 60.26 Mb (approximately 10 Mb) based on physical map distance from J 2658 in to ISCB 29004. This region contains component trait alleles such as QTdl.dsr-1.1 (Satish et al., 2009, 2012), QSv.dsr-1.1; QGs.dsr-1 (Aruna et al., 2011a), QEg28.dsr-1.1 (Satish et al., 2012), and QSv (Apotikar et al., 2011). Upon observing the ILs screening data of the cross ICSB 29004 × J 2658, the trichome density (**Figure 4C**) for most of the progenies was higher compared to the recurrent parent in both the seasons *viz*., 2014 post-rainy as well as in 2015 rainy. Two progenies showed higher seedling vigor (**Figure 4B**) than recurrent parent in 2014 post-rainy season whereas all the progenies showed lower vigor compared to recurrent parent in 2015 rainy season. During both the seasons, glossy character was observed among all the progenies (**Figure 4A**). And the scores for glossiness were either higher or similar to that of the recurrent parent. In case of oviposition, four progenies showed lower egg-laying percentage than recurrent parent during 2014 post-rainy and 2015 rainy seasons, of which two progenies (6018-5 and 6018-25) were common for both the seasons (**Figure 4D**). The overall dead-heart percentage among the ILs in this cross showed that two progenies (6018-5 and 6018-25) performed consistently better in both the screening seasons (**Figure 4E**).

### Phenotyping of Introgression Lines from Cross ICSB29004 × J2714

**FIGURE 5:**
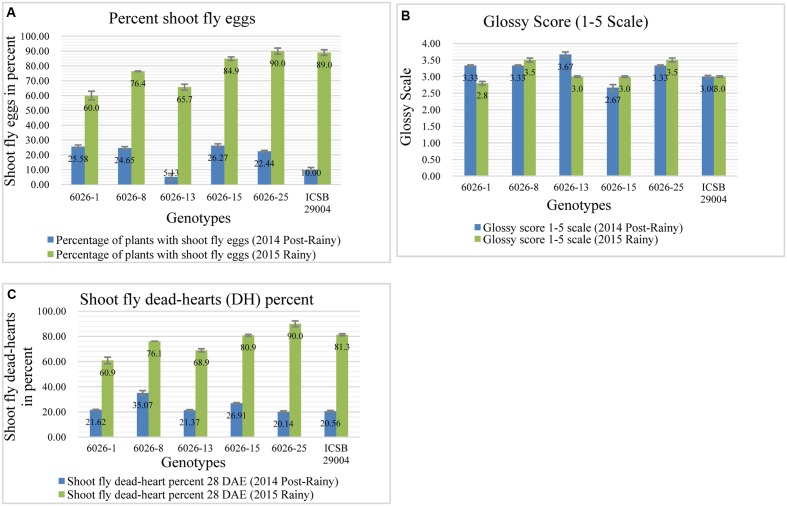
Performance of different introgression lines (1–5) along with their recurrent parent (ICSB 29004) for different shoot fly resistance component traits in two screening seasons. **(A)** Percentage of shoot fly eggs compared to recurrent parent, **(B)** Glossiness score for introgression lines compared to recurrent parent. **(C)** Dead-hearts percent when compared to recurrent parent. Bars represents standard error. Significance was determined at *P* < 0.05.

Here we transferred a QTL region on chromosome SBI-07 between 45.2 and 51.1 Mb based on physical map distance. It was reported that this region contributes to the alleles for oviposition non-preference, QEg21.dsr-7; QEg28.dsr-7 (Satish et al., 2009) glossiness, QGs.dsr-7 (Satish et al., 2012), and for dead hearts, Qdh.dsr-7.1; Qdh.dsr-7.2 (Aruna et al., 2011a). Among the five selected progenies having this QTL region, only one progeny (6026-13) showed lower percentage of shoot fly eggs (**Figure 5A**) than recurrent parent in 2014 post-rainy season whereas three progenies 6026-1, 6026-8, and 6026-13 showed lower shoot fly eggs in 2015 rainy season. The same trend was followed for all three progenies for dead-hearts percent when compared to recurrent parent at 28 DAE in 2015 rainy season (**Figure 5C**). One progeny, i.e., 6026-13 was common in both the seasons for having lower percent of shoot fly eggs. Four progenies in 2014 post-rainy and three progenies in 2015 rainy season showed glossiness more than recurrent parent (**Figure 5B**).

Performance of different introgression lines along with their recurrent parent Parbhani Moti (SPV 1411) for different shoot fly resistance component traits in two screening seasons. Bars represents Standard error. Significance was determined at *P* ≤ 0.05.

### Phenotyping of Introgression Lines from Cross Parbhani Moti (SPV 1411) × J2614

**FIGURE 6:**
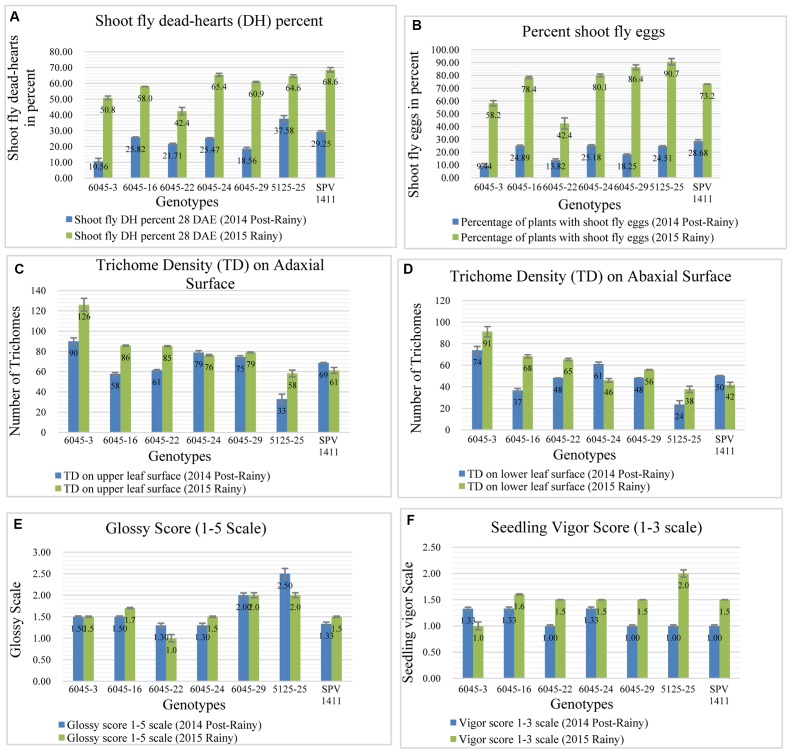
Performance of different introgression lines along with their recurrent parent Parbhani Moti (SPV 1411) for different shoot fly resistance component traits in two screening seasons. **(A)** Lower dead hearts percent compared to recurrent parent, **(B)** Percentage of plants with shoot fly eggs in both the seasons, **(C,D)** Trichomes on upper and lower leaf surface compared to recurrent parent, **(E)** Glossiness showing higher glossiness than recurrent parent during both the seasons, **(F)** seedling vigor compared to recurrent parent in both screening seasons. Bars represents standard error. Significance was determined at *P* < 0.05.

In case of third cross, approximately a region of 6 Mb, from 54.4 to 60.5 Mb has been transferred from chromosome SBI-10. This region contains the alleles for major shoot fly resistance component trait such as for dead hearts, Qdh.dsr-10.1; Qdh.dsr-10.2; Qdh.dsr-10.3; Qdh.dsr-10.4 (Deshpande, 2005; Satish et al., 2009, 2012; Aruna et al., 2011a), oviposition non-preference QEg21.dsr-10; QEg28.dsr-10 (Satish et al., 2009, 2012), trichome density on upper and lower surface of the leaf (designated as Tdu and Tdl) QTdu.dsr-10.1; QTdl.dsr-10.1; QTdu.dsr-10.2; QTdl.dsr-10.2 h (Deshpande, 2005; Satish et al., 2009, 2012; Aruna et al., 2011b), Glossiness QGs.dsr-10 (Satish et al., 2009, 2012; Aruna et al., 2011a) and seedling vigor QSv.dsr-10 (Satish et al., 2009, 2012). Out of six selected progenies five progenies showed lower dead hearts percent in 2014 post-rainy season and in 2015 rainy season two progenies showed lower dead hearts percent when compared to recurrent parent (**Figure 6A**). The same trend was seen in percentage of plants with shoot fly eggs in both the seasons (**Figure 6B**). The trichomes were better expressed in 2015 rainy season where out of six crosses five crosses showed more trichomes both on upper and lower leaf surface than the recurrent parent (**Figures 6C,D**). In contrast, only two crosses showed higher trichomes than recurrent parent in 2014 post-rainy season and four crosses showed higher trichomes in 2015 rainy season. Out of six crosses one cross showed consistently higher trichomes in both the seasons. In case of glossiness, two progenies showed higher glossiness than recurrent parent during both the seasons (**Figure 6E**). All progenies showed similar seedling vigor as that of recurrent parent in both screening seasons (**Figure 6F**).

The differences observed between ILs and parents for the data recorded on shoot fly resistance traits were subjected to statistical analysis. The ANOVA showed variation among the ILs (from three different crosses) and their respective recurrent parents for all shoot fly traits in two screening seasons (2014 post-rainy and 2015 rainy). The best ILs (two lines) from each cross showed significantly better shoot fly resistance compared to the recurrent parent. All shoot fly component traits contribute to the dead hearts percentage. The best plants, which are consistent and showed low dead-hearts percentage in both the screening seasons were selected. For the cross ICSB 29004 × J 2658, two ILs *viz*., 6018-5 and 6018-25 showed low dead hearts percentage. Also, these two selected lines exhibited 80 and 91.6% recurrent parent genome, respectively. For the cross ICSB29004 × J2714, two ILs viz., 6026-1 and 6026-13 with low dead hearts and recurrent parent genome recovery of 84 and 88.9%, respectively were selected. Lastly, for the cross Parbhani Moti (SPV 1411) × J2614, ILs 6045-3 and 6045-22 showed lower dead hearts percentage and with recurrent parent genome recovery of 81.3 and 87.5%, respectively. During selection process, preference was given to 2015 rainy season data as the shoot fly population pressure was high.

### Agronomic Performance of QTL Introgression Lines

**FIGURE 7:**
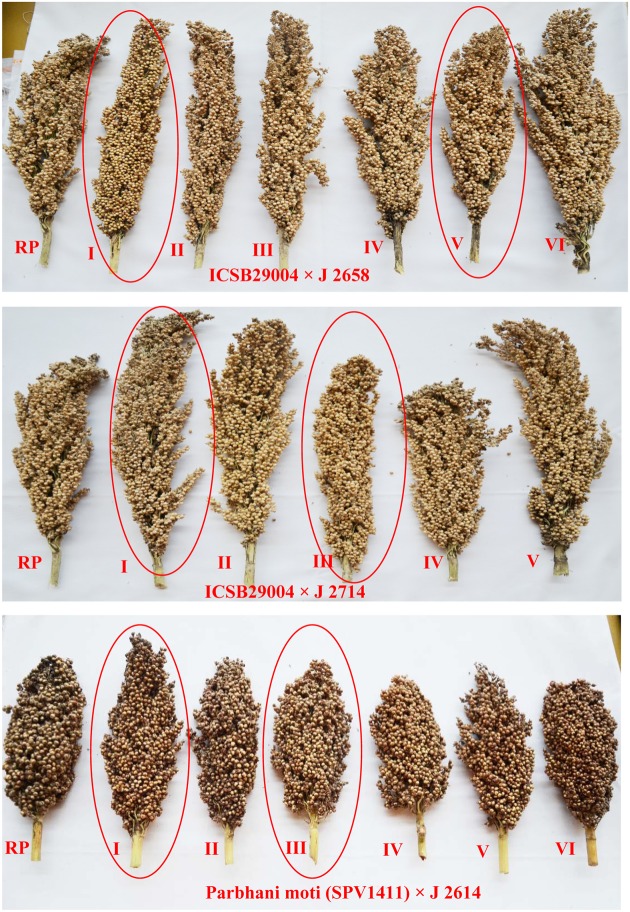
Mature sorghum panicles of introgressed lines along with recurrent parents. Panicles in red circles are selected lines for shoot fly resistance.

The agronomic data of ILs were collected for traits *viz*., days to 50% flowering, 100 grain weight, panicle weight and grain weight from all three crosses which showed similar or higher yield performance of ILs compared to the recurrent parent (**Table 6**). Days to 50% flowering remained constant between the ILs and their respective recurrent parents. The six selected ILs exhibited better grain yield along with higher shoot fly resistance (**Figure 7**). These ILs were also superior to recurrent parents in terms of shoot fly resistance. The ILs were phenotypically similar as that of recurrent parent. The donor parent though carrying the resistant QTL did not performed well.

**Table 6:** Agronomic performance of different introgression lines in comparison to their recurrent parents in two 2014 Post-rainy (PR) and 2015 Rainy (R).

		Days to 50% flowering		100 Seed weight (g)		Grain weight (g)		Plant Aspect score (1 = best and 5 = poor)	
S. No	Genotypes	2014PR	2015R	2014PR	2015R	2014PR	2015R	2014PR	2015R
1	6018-5	68	77	2.8	2.6	485	920	1	1
2	6018-12	68	78	2.7	3.1	795	663	2	2
3	6018-15	66	81	2.6	2.9	707	892	1	2
4	6018-20	66	77	1.7	3.0	453	773	2	2
5	6018-25	66	79	2.7	3.2	483	936	1	2
6	5135-8	70	81	3.0	2.7	297	764	1	2
7	ICSB29004 (RP)	72	81	3.0	2.8	425	865	2	2
8	J-2658 (DP)	65	75	2.6	2.0	118	483	4	4
9	6026-1	69	81	3.0	1.6	365	739	1	1
10	6026-8	69	81	2.9	2.7	592	847	2	2
11	6026-13	66	81	2.6	2.7	297	988	1	2
12	6026-15	68	82	2.7	2.7	542	811	1	1
13	6026-25	69	81	3.2	3.0	433	495	1	2
14	ICSB29004 (RP)	72	81	3.2	3.3	295	709	2	2
15	J-2714 (DP)	68	79	2.9	2.4	113	541	3	3
16	6045-3	66	81	3.8	2.6	289	663	1	1
17	6045-16	69	81	3.6	2.7	202	758	2	2
18	6045-22	66	77	3.8	2.4	260	627	1	1
19	6045-24	66	79	4.2	1.9	302	505	2	2
20	6045-29	67	80	3.9	2.5	200	818	1	1
21	5125-25	72	82	3.6	1.9	117	465	2	2
22	SPV 1411(RP)	72	81	5.0	2.9	238	607	2	2
23	J-2614 (DP)	65	74	2.9	2.2	102	596	3	3
24	IS18551 (res check)	71	79	3.2	1.8	698	902	1	2
25	296B (sus check)	70	82	2.9	3.0	353	891	1	3

*S. No 1–6 are Introgression Lines and S. No 7–8 are their corresponding recurrent parent (RP) and donor parents (DP). Similarly, S. No 9–13 are introgression lines and S. No 14–15 are their corresponding parents and S. No 16–21 are Introgression Lines and S. No 22–23 are their corresponding parents. Grain weight is weight of sorghum grain per plot average dof three replications*.

## Discussion

Shoot fly is one of the leading threat for sorghum production, globally, causing severe yield losses. Progress has been made in achieving shoot fly resistance using classical breeding methods, however, these methods are labor-intensive and time-consuming (Sharma et al., 2003). To support and increase the efficiency of conventional breeding, deploying molecular markers linked to QTLs or any gene, governing the trait of interest and transferring of these QTLs/gene is advocated (Kumar et al., 2013). This approach can be used to generate cultivars with desired characters in less time and high precision (Varshney et al., 2010). In the present study, shoot fly resistance QTLs were introgressed in genetic backgrounds ICSB 29004 and Parbhani Moti. Both the recurrent parents selected were agronomically elite and preferred by the farmers and researchers. Parbhani Moti is most popular post-rainy season sorghum cultivar prized for its grain and stover yield and quality and ICSB 29004 is elite female parent used in hybrids production. To reduce the linkage drag associated with shoot fly resistance donor landrace germplasm IS 18551, three elite BTx623 derivatives carrying shoot fly resistance QTLs (introgressed earlier from IS 18551) were used as donor parents.

Based on different studies made earlier on shoot fly resistance, 29 QTLs showed association with shoot fly resistance component traits (Satish et al., 2009). These QTLs were present on SBI 01, SBI 07, and SBI 10 chromosomes and control the component traits *viz*., trichome density, seedling vigor, glossiness, oviposition non-preference and dead hearts percentage. Considering this, the three QTLs conferring resistance to shoot fly were targeted for introgression into the recurrent parents, ICSB 29004 and Parbhani Moti.

Knowledge of parental polymorphism is a pre-requisite to initiate any backcrossing program. Polymorphic parents helps in selection of plants carrying the trait of interest in progenies in each generation. The parents used in this crossing program belongs to different racial backgrounds with diverse geographic origins. The recipient parent ICSB 29004 has *caudatum* and *durra* race germplasm accessions originated in India in its pedigree whereas the QTL donors (J2658, J2614, and J2714) are derivatives of BTx623 from United States. Parbhani Moti is a selection from *durra* landrace accession in India. Therefore the diversity among the parents is higher which manifested in the form of polymorphism. Also, studies from past 20 years on molecular marker variation in sorghum showed less genetic similarity between the landraces from same geographic region and accessions from the same race but having distinctly different geographic origins (Hash, 2010). In our earlier study, a similarity index obtained using for 33 SSR loci ranged from 0.1 to 1.0 (Gorthy et al., 2016). In the current study, for two crosses, ICSB29004 and J 2658, and SPV 1411 and J 2614 the similarity index was 0.2 and 0.1 indicating the diversity among parents. Cluster analysis based on similarity values showed that the donor and recurrent parents were present in different clusters.

Simple sequence repeat markers with a high polymorphism contributes many agronomically important traits in sorghum. Genomic SSRs shows high polymorphism and are distributed throughout the genome (Kuleung et al., 2004). Mutations occurred during evolution could be one of the reason for high rate of polymorphism (Nei, 2007). It was reported that, genomic SSRs and EST-SSRs with di and tri -nucleotide repeats exhibit high polymorphism compared to the other nucleotide repeats. In sorghum, variation in mutation within genomes has been correlated with varying rates of recombination and di- or trinucleotide repeats (Michaelson et al., 2012). Another reason for the presence of trinucleotide repeats in coding regions may be the exertion of selection pressure for selecting single amino acid (Morgante et al., 2002). Studies in cotton, showed genomic SSRs exhibit more alleles in microsatellite regions hence they can be used for fingerprinting and for the estimation of genetic diversity (Tabbasam et al., 2014) It is predicted that polymorphism is influenced by mutation factors and selection pressure and vary according to the local recombination rate in wheat (Thuillet et al., 2004). Studies show that mutation rates are higher in high recombination region showing positive correlation between nucleotide diversity and recombination percent (Marais et al., 2001; Lercher and Hurst, 2002). The sorghum chromosomes contain distal euchromatic regions which is high in DNA polymorphism and large pericentromeric region with low gene density and recombination (Evans et al., 2013). The microsatellites located in distal regions had longer alleles than loci in centromeric regions. As longer alleles are expected to have comparatively higher mutation rates, distal regions should generate more mutant alleles at microsatellite loci, and consequently, should be more polymorphic. As the polymorphism is observed in the parents, the SSRs present in the QTL region or flanking the QTL region can be used as FGS markers.

Foreground selection was done with QTL-linked markers to identify the heterozygotes in all the three crosses and also to select heterozygotes in each backcross generation for further backcrossing or selfing. For selection of plants with maximum recurrent genome, SSR markers spanning all the 10 sorghum chromosomes were used. Two backcrosses and three selfings were made to have good recovery of recurrent parent genome and to make the lines homozygous for most of the loci.

Out of six plants in BC_2_F_4_ generation of the cross ICSB 29004 × J 2658, five were identified with recurrent parent genome with a recovery percentage ranging from 80 to 92% in comparison to the 87.5% of expected average similarity. Similarly, in the case of ICSB 29004 × J2714 and SPV 1411 × J2614, 5 and six plants, respectively were identified with 80 to 90% of recurrent genome. Fixation of the heterozygous alleles might be the reason behind less recurrent genome recovery in some of the selected plants in BC_2_F_1_ generation toward donor parent genome (Varshney et al., 2014). However, the plants were selected considering the overall performance in shoot fly screening block along with recurrent parent genome recovery. This gives more strength to the selection process as both genotypic and phenotypic information is combined in the selection process. The three crosses were analyzed for lesser donor parent chromosomal segments. For this, carrier chromosome specific polymorphic SSR markers were used and analyzed the recovery of recurrent parent alleles on carrier chromosomes in BC_2_F_4_ ILs.

Phenotyping of MABC derived ILs and parental lines for shoot fly resistance in screening block using interlard fish meal technique showed resistance reaction in some lines. The selected six progenies from three crosses exhibited better shoot fly resistance, manifested by lower dead hearts percentage compared to their respective recurrent parents indicating that transferred QTLs were effective in contributing for shoot fly resistance. In earlier reports the major QTLs for glossiness on SBI-05 and SBI-10 (QGs.dsr-10) explaining 14 and 14.7% of the phenotypic variation, respectively and a minor QTL on SBI-01 explaining less phenotypic variation of 5.9% were identified as some effect on shoot fly resistance (Satish et al., 2009; Aruna et al., 2011a). In our study, the expression of leaf glossiness was found higher in all the six progenies than their respective recurrent parent leading to lower oviposition and dead heart percentage.

Leaf glossiness plays a significant role in reflecting the light which reduces the shoot fly population around the seedlings, resulting in reduction in oviposition (Sharma, 1993). Higher glossiness reduces the shoot fly infestation. In this study, all the six crosses showed a negative reaction between percentage of dead hearts and leaf glossiness, i.e., the plants with higher glossy score will have lower dead hearts (Nwanze et al., 1990; Kamatar and Salimath, 2003). Two QTLs for trichome density (upper and lower surface of leaves) were identified on chromosome SBI 10 and one QTL on lower surface only was identified on SBI 01. In both the cases the trichomes were more than the recurrent parent indicating the significance of trichomes presence in reducing the shoot fly dead hearts percentage. It was observed that there is a negative correlation between trichome density on leaves and oviposition and dead hearts (Sandhu et al., 1988; Dhillon et al., 2005a). Higher the trichomes lower is the oviposition thus reduction in dead hearts percent and *vice- versa*. In the ILs derived from cross Parbhani Moti × J2614 though the trichome density was high for few progenies, the dead hearts percentage did not change. The reason could be the trichome morphology (pointed unicellular vs. blunt bicellular trichome). Also, the presence of epicuticular wax which hinders the shoot fly to adhere on leaves may play a major role in restricting the oviposition. In previous studies, unicellular pointed trichomes were observed in resistant sorghum genotypes while the susceptible genotypes possess bicellular blunt trichomes (Padmaja et al., 2010a,b; Aruna et al., 2011a). Another important trait for shoot fly resistance in sorghum is seedling vigor. Some crosses in this study showed the vigor score was higher in the post-rainy season compared to rainy season in the crosses, ICSB 29004 × J 2658 and Parbhani Moti × J2614. As a result, the dead heart percentage was also low in 2014 post-rainy season than 2015 rainy season. Since one of the recurrent parents, Parbhani Moti is highly adapted to the post-rainy season and rapid seedling growth was observed. Fast growth slows down the larvae from reaching the central growing leaf, thereby reducing the chance of dead heart formation. Earlier studied have identified a gene (Leucine-rich repeat transmembrane protein kinase) responsible for meristem growth and defense (Dievart and Clark, 2004). Whereas seedlings showing slow growth rate or poor vigor are more vulnerable and succumb to shoot fly damage (Taneja and Leuschner, 1985). All the shoot fly component traits leads to a primary mechanism of non-preference to oviposition (Jotwani et al., 1971; Taneja and Leuschner, 1985; Sharma et al., 2003; Riyazaddin et al., 2016). Although the major QTL for ovipositional non-preference was identified on SBI-05, other minor QTLs on other chromosomes such as SBI 01, SBI 07 and SBI 10 also contributes to shoot fly resistance. In this study, two progenies from each cross showed consistently low egg count resulting in lower dead hearts percentage indicating the importance of oviposition non-preference to increased shoot fly resistance in sorghum. These traits need to be more exploited in sorghum improvement. Phenotyping of the ILs for agronomic traits indicated that some of the ILs showing shoot fly resistance is far superior to their recurrent parent for grain yield without any change in flowering time. This clearly showed the development of sorghum lines with shoot fly resistance without affecting grain yield can be achieved by using marker-assisted backcrossing. The identified lines can be further tested in multi-location field trials for commercialization in adapted locations. However, to add value further, the best MABC lines developed in the present study can be intercrossed to pyramid these QTLs for further increasing the shoot fly resistance.

In the present study, the SSRs are found to be highly polymorphic exhibiting different alleles among closely related individuals for each marker in relatively lesser time and minute quantity of DNA which is in agreement with recent reports in pigeonpea (Bohra et al., 2015, 2017). The SSR has been employed extensively in sorghum to study genetic diversity, linkage mapping and QTL analysis (Njung’e et al., 2016). Some studies showed that SNP markers shows better genetic relatedness with more population number whereas at the diversity level SSR markers are better for grouping of samples at trait level (Singh et al., 2013). The SSRs are the preferred type of molecular markers because of their abundance and amenability to high throughput screening whereas SNP markers should be preferably used for determination of population structure in crops.

## Conclusion

The present study suggests that SSR markers linked to QTLs controlling component traits for shoot fly resistance are reliable for marker-assisted backcrossing. The development of ILs using marker assisted backcrossing was comparatively faster than with conventional breeding. The recovery of the recurrent parent genome was close to 90% using MABC. Six improved shoot fly resistance ILs were produced from three crosses involving elite parental lines ICSB 29004, Parbhani Moti (SPV1411) and BTx623 derivatives (J2658, J2714, J2614) by subsequent backcrossing and selfing using foreground and background selection. The ILs showed higher shoot fly resistance and better grain yield with similar flowering time. These ILs can be commercialized after multi-location testing in adapted environments and can be used as a source of genetic material for improving shoot fly resistance in high yielding backgrounds. Development of novel resistant lines will lead to populating durable shoot fly resistant sorghum cultivars which will have a great impact on the yield stability and sustainability. To our knowledge, this is the first report on the successful introgression of shoot fly resistance QTLs into the elite sorghum cultivars ICSB 29004 and Parbhani Moti.

## Author Contributions

SG: PI of the Project; executed the research and made major contribution to developing the manuscript; LN: supervised the progress of research; AG: helped in field experimentation and phenotyping; HS: field phenotyping for shoot fly resistance; AK: helped in manuscript development; SD: planning of experiments, molecular biology support, genotyping data analysis; AA: Scientist –Mentor of SG; Planned the experiments, supervised the progress of research and contributed in preparing the manuscript.

## Conflict of Interest Statement

The authors declare that the research was conducted in the absence of any commercial or financial relationships that could be construed as a potential conflict of interest.
